# Innovative Approaches to Addressing Addiction in Real-World Clinical Settings

**DOI:** 10.1192/j.eurpsy.2025.852

**Published:** 2025-08-26

**Authors:** A. K. Sikora, S. T. Kayahan, M. Dzis, J. Dąbrowska

**Affiliations:** 1 Psychiatry, Addictions and Medical Psychology Department, Ivano-Frankivsk National Medical University, Ivano-Frankivsk, Ukraine; 2 HOSPITAL OF THE MAZOWIECKIE VOIVODESHIP DREWNICA, Warsaw, Poland; 3 Turkish Republic Ministry of Health Yalvaç State Hospital, Isparta, Türkiye; 4 Psychiatry, Psychology and Sexology, Danylo Halytsky Lviv National Medical University, Lviv, Ukraine; 5 Szpital Bielański im. Ks. Jerzego Popiełuszki, Warsaw, Poland

## Abstract

**Introduction:**

Addiction is a complex condition affecting millions globally, involving behaviors from substance use to behavioral addictions like gambling disorder (GD). It frequently co-occurs with psychiatric disorders, such as bipolar disorder (BD) and bulimia, complicating treatment. Individuals with BD are six times more likely to develop GD, especially men. This presentation explores managing various forms of addiction across different populations.

**Objectives:**

1. Provide updated knowledge on addiction and its comorbidities.
2. Explore integrated treatment for co-occurring psychiatric disorders.
3. Highlight the role of cultural contexts in addiction treatment.
4. Equip clinicians with practical tools for comprehensive addiction care.

**Methods:**

Four key areas of addiction management are discussed using case studies and comparative analysis:

1. Compulsive Sexual Behavior in Veterans: Examining compulsive sexual behavior as a PTSD symptom, diagnosis, and treatment approaches.
2. Polish Addiction Treatment Standards: A comparative analysis of Polish standards and recent reforms, and their impact on practice.
3. Dual Diagnosis: Alcohol Addiction and Bulimia: Case-based discussions on integrating treatment for co-occurring alcohol addiction and bulimia.
4. BD and GD Comorbidity: Reviewing pharmacological (e.g., lithium) and psychosocial interventions for BD-GD comorbidity.

**Results:**

1. Compulsive Sexual Behavior in Veterans: Tailored interventions improve outcomes for veterans.
2. Polish Addiction Standards: Reforms improve accessibility, but aligning with European standards for dual diagnosis remains challenging.
3. Dual Diagnosis: Integrated treatment for alcohol addiction and bulimia improves outcomes and reduces relapse rates.
4. BD and GD Comorbidity: Lithium and psychosocial interventions stabilize mood and reduce gambling, but more research is needed.

**Conclusions:**

Innovative approaches to addiction management, particularly for co-occurring psychiatric disorders, improve clinical outcomes. Tailored interventions for veterans, analysis of treatment standards, and integrated strategies for dual diagnoses provide practical solutions for clinicians. Further research is needed to optimize treatments for BD-GD comorbidity.

**Disclosure of Interest:**

None Declared

